# Morphological classification of the temporalis muscle: anatomical, radiological, and surgical perspectives

**DOI:** 10.3389/fsurg.2026.1704668

**Published:** 2026-03-03

**Authors:** Adrian Okoń, Ingrid C. Landfald, Łukasz Olewnik

**Affiliations:** 1 Department of Clinical Anatomy, Mazovian Academy, Płock, Poland; 2 VARIANTIS Research Laboratory, Department of Clinical Anatomy, Mazovian Academy in Płock, Płock, Poland

**Keywords:** anatomical variation, classification, deep temporal fascia, magnetic resonance imaging, orthognathic surgery, temporalis muscle, temporalis tendon, ultrasound

## Abstract

The temporalis muscle (TM) shows substantive morphological variability with direct implications for surgical planning and imaging interpretation. Drawing on cadaveric series and imaging cohorts, we critically synthesise convergent findings on layered architecture, distal tendon configuration and retromolar extensions, and highlight discrepancies largely attributable to plane/sequence selection and operator dependence in magnetic resonance imaging and ultrasound. We propose a four-type classification with operational criteria based on fascicular architecture, terminal tendon pattern and accessory slips, including relationships to the deep temporal fascia and the temporomandibular joint capsule. This scheme should be regarded as a pragmatic, hypothesis-generating framework derived from heterogeneous descriptive datasets rather than a statistically validated taxonomy. Where available, we summarised reported frequencies of Types II–IV across cadaveric and imaging cohorts using a study-level evidence map. We further translate type-specific anatomy into decision support for flap harvest and lengthening temporalis myoplasty, osteotomy/coronoidectomy planning, targeted management of temporomandibular disorders (e.g., botulinum toxin, dry needling), and radiotherapy contouring. Recognising temporalis variability can improve diagnostic accuracy, optimise operative strategy and reduce complications, while underscoring the need for harmonised definitions and prospective validation.

## 1 Introduction

### 1.1 Significance of the temporalis muscle in functional and clinical anatomy

The temporalis muscle (TM) is a principal elevator of the mandible that also contributes to retrusion and stabilisation during complex jaw movements (1, 2). Arising broadly from the temporal fossa and inserting into the coronoid process, the TM generates substantial bite force while presenting layered architecture and distal configurations relevant to surgical planes (3). These anatomical relations underpin reconstructive and orthognathic approaches and influence management of temporomandibular joint disorders (TMD) (4, 5). Clinically, the TM is implicated in myofascial pain and bruxism, and iatrogenic changes may lead to temporal hollowing or functional deficits (6). Its bulk and accessibility also enable reliable regional flaps, provided anatomical integrity is respected, and its role in sensorimotor control supports proprioceptive fine-tuning of mastication (7, 8).

### 1.2 Underappreciated morphological variability and its impact on diagnosis and therapy

Despite its recognised functional importance, the TM's morphological variability remains underappreciated in clinical and radiological practice (9, 10). Variation in the number of bellies, the extent/configuration of tendinous insertions, and accessory slips or fascial expansions can alter mechanics, complicate imaging interpretation and reshape surgical planning (4, 11). On magnetic resonance imaging (MRI) and ultrasound (US), fine accessory slips or temporomandibular joint (TMJ)-related bundles may be under-detected, risking misclassification or inappropriate treatment (6, 9). Intraoperatively, unrecognised variants increase the likelihood of nerve injury, compromise flap perfusion and affect functional and aesthetic outcomes (5). Accordingly, a precise understanding of TM variability is essential to improve diagnostic accuracy, enhance surgical safety and individualise therapy (10, 12–14).

#### 1.2.1 Contribution of this review

We provide a study-level evidence map to align anatomical and imaging descriptions using harmonised terminology, propose operational definitions for a four-type, side-level classification, and summarise imaging correlates and procedural implications to support reproducible reporting and future prospective validation.

The aim of this review is to synthesise the reported morphological variability of the temporalis muscle, integrating anatomical descriptions, MRI and ultrasound correlates, and procedural implications. We further propose operational, hypothesis-generating criteria for a four-type classification framework intended to organise existing evidence and to facilitate prospective validation, rather than to estimate prevalence or establish outcome-validated recommendations. We conducted a structured literature search and evidence mapping as described below.

## 2 Methods

### 2.1 Protocol and reporting

This structured narrative review focuses on the TM, its tendon/aponeurotic layering, and clinically relevant variants that may influence reconstructive and orthognathic procedures. Methods were specified *a priori* and reporting follows best practice for structured narrative reviews (including PRISMA-S elements where applicable). No protocol was registered.

### 2.2 Information sources and search strategy

We searched MEDLINE (via PubMed) and Embase from database inception to 11 November 2025 (humans; English). Searches combined controlled vocabulary and free-text terms for temporalis muscle anatomy and variation, tendon/aponeurotic and fascial lamination, imaging (MRI/ultrasound), and surgical relevance. Full executable database strategies and limits are provided in the Supplementary Appendix (Supplementary Table S1). Because the imaging component of the core string was optimised for MRI/ultrasound terminology, we additionally performed backward reference screening and forward citation tracking of key articles to capture relevant records not consistently retrieved by the core query (including CT/CBCT series and intraoral/interventional ultrasound papers). Reference lists of eligible studies and key reviews were hand-searched for additional records. No study-design or date filters were applied beyond language and species limits.

### 2.3 Eligibility criteria

We included human studies (cadaveric dissections, imaging studies, surgical series, and clinically oriented case reports/series) that met at least one of the following: (1) described TM morphology and/or deep/superficial temporal fascia and tendon/aponeurotic layering; (2) defined a TM variant using operational anatomical or imaging criteria; or (3) linked such variants to clinical decision-making or surgical planning. We excluded animal-only studies; methodological or technical notes without primary anatomical/imaging observations; narrative opinion pieces without extractable data; and works unrelated to the temporalis region. Articles required an English abstract; full texts had to be available in English.

### 2.4 Study selection

Two reviewers independently screened titles/abstracts and then full texts against the criteria above. Disagreements were resolved by discussion; a senior author adjudicated when necessary. Reasons for full-text exclusions were recorded (e.g., non-TM focus, insufficient definition of the variant, no extractable observations). Duplicates were removed prior to screening.

### 2.5 Data extraction

From each study we extracted: setting/design; modality (cadaver/MRI/ultrasound/other); sample size; operational definition of the variant; any study-level frequencies (counts/percentages with denominators); key anatomical features (belly count, fascial lamination, tendon footprint, accessory slips and their course); imaging planes/sequences; and clinical notes (surgical relevance or intra-operative implications). Extraction used a predefined template aligned with our Evidence Map (Table 1).

**Table 1:** Evidence map of temporalis muscle variants (study-level data).

Author	Year	Country/Population	Modality	N	Operational definition	Reported frequency (*n*/*N*, %)	Key anatomical features	Imaging planes/sequences	Quality notes	Contextual notes	Mapped to proposed type(s)
Geers et al.	2005	Belgium; adult cadavers	Cadaveric dissection with MRI correlation	10 cadavers (20 sides)	Deep belly defined as a deep portion of temporalis without epimysial separation, correlated with MRI.	10/10 (100%)	Deep portion contiguous with temporalis; adjacent to lateral pterygoid; not a separate muscle.	T1-weighted MRI correlation	Operational definition supported by histology and MRI correlation.	Supports interpretation as internal architecture rather than a distinct muscle entity.	Type I component (internal architecture or deep bundle concept)
Sedlmayr et al.	2009	USA; cadavers and clinical cohort	Cadaveric dissection with CT/MRI corroboration	16 cadavers (32 sides) plus 10 patients	Temporalis described as superficial, zygomatic, and deep parts comprising one structural unit; occasional atypical superficial extension documented.	16/16 cadavers (100%) tripartite description; 1/16 cadavers (6%) atypical superficial extension; patient cohort used for radiologic corroboration without prevalence estimates.	Deep fibres related to inner coronoid/retromolar region; zygomatic part from zygomatic arch joins superficial portion.	CT and T1-weighted MRI in clinical cohort	Combined anatomical and radiologic dataset; clear part-based description.	Defines multi-part descriptions within one muscle and documents occasional atypical extension.	Type II component (partitioning into intrinsic parts); supports overlap concept used in Type IV
Lee et al.	2012	South Korea; adult cadavers	Cadaveric dissection with histology and MRI confirmation	20 heads	Distinct superficial layer separable from deep layer; superior intermingling described.	20/20 (100%)	Superficial vs. deep lamination with overlapping origins; layered configuration.	MRI confirmation (planes not specified)	Dissection, histology, and MRI confirmation; focused sample with explicit terminology.	Defines layered architecture and terminology relevant to lamination.	Type I component (layered architecture)
Benninger and Lee	2012	USA; embalmed cadavers	Cadaveric dissection	30 cadavers	Anterior distal tendon extending onto the retromolar triangle/fossa defined by landmark-based criteria.	30/30 (100%)	Insertion along medial and lateral borders of retromolar triangle; extension to anterior mandibular ramus.	NR	Operationalised landmark-based description; consistent observation across specimen set.	Documents substantive distal extension beyond the usual footprint.	Type III component (retromolar or accessory distal attachment)
Prasidha et al.	2020	Australia; fresh-frozen cadavers	Cadaveric dissection (surgical simulation)	12 cadavers (24 muscles)	Accessory attachments near the main insertion mapped relative to posterior deep temporal artery.	NR	Multiple accessory attachments near insertion; relationships relevant to release and pedicle preservation.	NR	Fresh tissue with clear measurements and surgical-relevant mapping.	Documents accessory attachments near insertion and their relationship to the main pedicle.	Type III component (accessory attachments near insertion)
Yu et al.	2021	South Korea; adult cadavers	Cadaveric dissection and morphometry	26 cadavers	Two terminal tendons with broad coronoid footprint; fascicle direction classification; distal tendon organisation described.	26/26 (100%)	Superficial tendon along lateral retromolar boundary and deep tendon medially; broad coronoid footprint.	NR	Operational mapping of distal tendon complex; single-centre series.	Defines dual terminal tendon configuration and distal footprint organisation.	Type I component (dual terminal tendon architecture); informs Type III only when an additional discrete accessory band beyond the usual footprint is explicitly described
Shimokawa et al.	1998	Japan; adult cadavers	Cadaveric dissection (innervation analysis)	5 cadavers (10 temporalis muscles)	Small muscle bundles attached to the temporalis defined by gross dissection, with intramuscular nerve supply traced to deep temporal nerve branches.	10/10 (100%) temporalis muscles had three small additional bundles described	Three small additional bundles adjacent to the temporalis; distinct innervation patterns suggest compartmentalisation and functional subdivision.	N/A	Descriptive cadaveric series; focused on innervation tracing rather than prevalence estimation.	Provides neuroanatomical evidence for accessory bundles/compartmentalisation within the temporalis region.	Type II component (multiple bundles/compartmentalisation); may contribute to Type IV when combined with accessory attachments
Kageyama and Itoh	2003	Japan; anatomical specimens	Cadaveric dissection	54 sides	Deep part described as a deep muscle bundle with a tendon of origin at the infratemporal crest; superficial and deep tendon layers (superficial tendon and deep tendon) were distinguished at the insertion.	Deep muscle bundle present in all specimens (54/54 sides); infratemporal crest processes noted in 49/54 (90.7%) sides	Deep muscle bundle oriented to the infratemporal crest; tendon layers at the distal insertion (superficial vs. deep) described; relationship to infratemporal crest morphology.	N/A	Detailed anatomical documentation; denominators reported per side; not an imaging cohort.	Supports operational separation of deep bundle and layered tendon concepts relevant to harmonised terminology.	Type I component (layered distal tendon); Type II component (deep bundle/partitioning)
Palomari et al.	2013	Brazil; cadaveric material	Cadaveric anatomical study	NR	Evaluates whether the sphenomandibularis corresponds to a distinct muscle or a deep bundle of temporalis based on continuity and anatomical relations.	NR	Deep bundle adjacent to the infratemporal region; continuity with temporalis emphasised.	N/A	Addresses a nomenclature and ontology controversy directly relevant to classification clarity.	Supports treating the deep bundle as temporalis internal architecture rather than a separate muscle.	Type I component (internal architecture or deep bundle)

Frequencies are reported as *n*/*N* (%) when available; otherwise NR (not reported) or N/A (not applicable). Denominators refer to specimens unless specified (sides or heads).

### 2.6 Quality appraisal

Given the lack of validated risk-of-bias tools for anatomical/imaging variation research, we applied a domain-based appraisal adapted to this topic: (1) clarity/reproducibility of variant definitions; (2) ascertainment(cadaveric technique; imaging protocol including field strength, coil, planes, and slice thickness); (3) sampling and representativeness; (4) observer processes (number of assessors, independence/blinding, inter-rater agreement where reported); and (5) completeness of reporting (use of standard anatomical terminology consistent with *Terminologia Anatomica*). Domains were qualitatively graded as *low risk, some concerns*, or *high risk*.

### 2.7 Synthesis methods

Owing to heterogeneity in definitions, imaging planes, and denominators, no meta-analysis was attempted. We present a comparative Evidence Map (Table 1) of study-level frequencies and features, and derive an operational four-type classification. Each type is then mapped to (a) imaging signatures and common pitfalls (with practical mitigation steps) and (b) concrete uses in surgical planning and clinical decision-making.

### 2.8 Terminology harmonisation

Throughout the review we normalised nomenclature to British English and Terminologia Anatomica: *temporalis muscle (TM)* after first use; *fossa temporalis*; *coronoid process*; *deep temporal fascia (DTF)*; *superficial temporal fascia (STF)*; and *frontal branch of the facial nerve (FBFN)*. Synonyms encountered in primary sources were reconciled to these terms during extraction to ensure comparability.

## 3 Embryological and developmental background

The TM originates embryologically from the mesenchyme of the first pharyngeal (branchial) arch, specifically the mandibular arch, which gives rise to the muscles of mastication (15, 16). The initial myogenic condensations emerge during the fifth to sixth week of gestation, guided by intricate signalling cascades and spatial cues (15–17). Differentiation of the deep temporal fascia (DTF) and superficial temporal fascia (STF) provides laminar envelopes that guide superficial–deep stratification and the distal tendon footprint (2).

Craniofacial myogenesis follows canonical programmes in which MYOD1 and MYF5 drive myogenic commitment, while Wnt/β-catenin and TGF-β/SMAD signalling shape patterning and tendon/fascial matrix formation (18–20).

During early myogenesis, muscle fibres within the TM (17) begin to segment into discrete bundles, which later correspond to functionally distinct regions or bellies (21). Concurrently, the differentiation of tendinous attachments establishes the foundational architecture for muscle insertion points, particularly on the temporal fossa and mandibular coronoid process (17).

Following the onset of jaw function, cranial growth and functional loading further refine TM architecture—mechanotransduction shapes final tendon layering and belly segmentation (22–25).

## 4 Documented morphological variants

### 4.1 Classical description of the TM and its tendon

**Figure 1:**
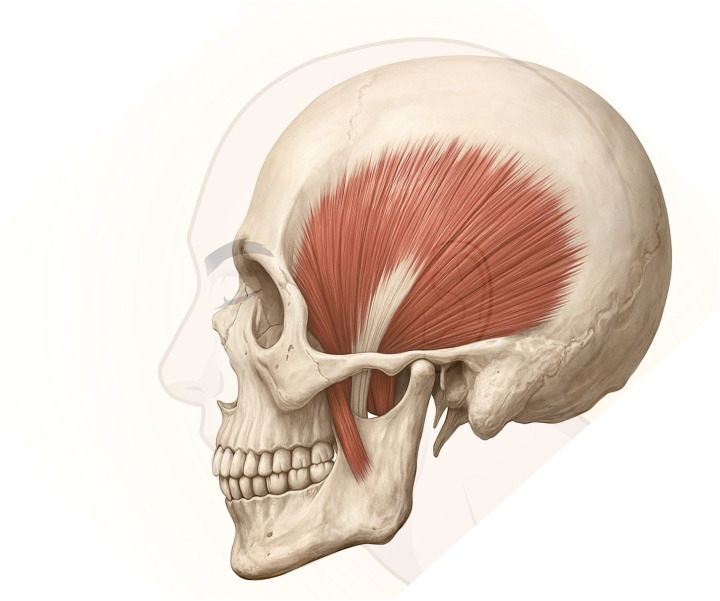
Temporalis muscle in lateral cranial view showing fan-like fiber arrangement and tendinous insertion on the coronoid process of the mandible.

**Figure 2:**
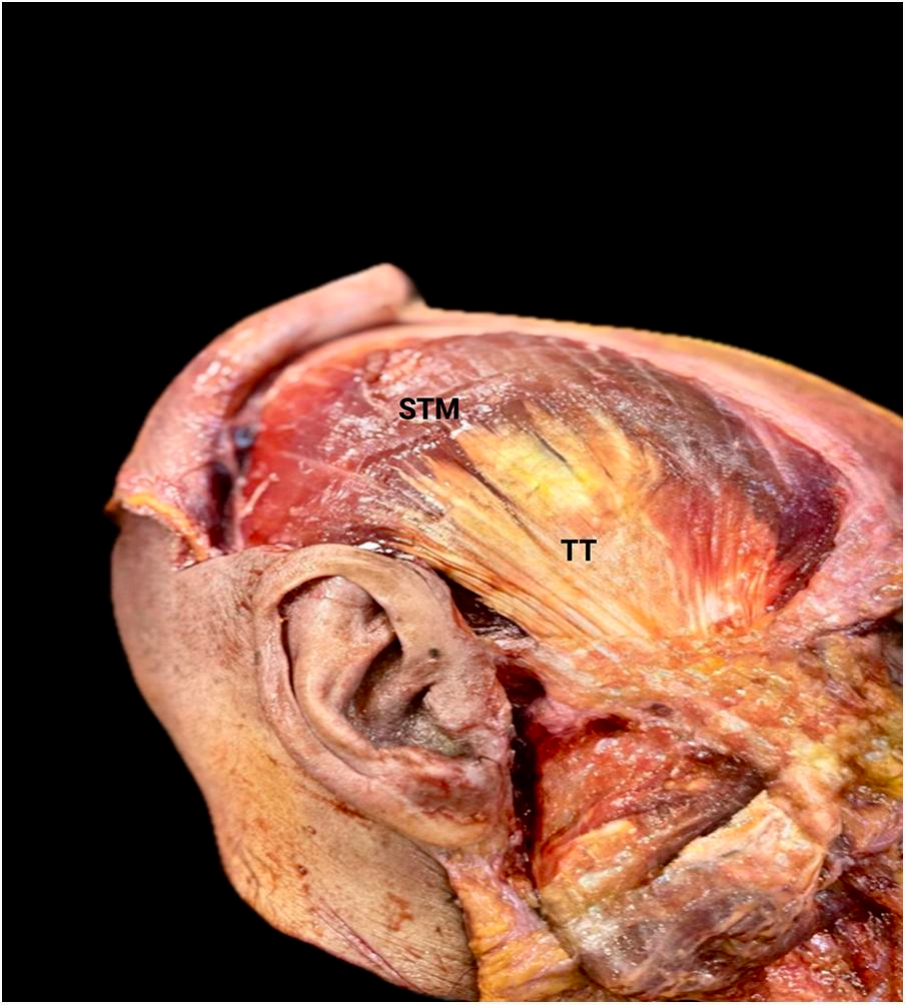
Cadaveric lateral view of the temporalis muscle showing the superficial portion (STM) and temporalis tendon (TT).

The TM is a broad, fan-shaped elevator of the mandible occupying the temporal fossa, with convergent fibres inserting on the coronoid process through a distal tendinous complex contained within the temporal fascial envelopes (1, 26–28). Figures 1, 2 illustrate the classical Type I configuration. Contemporary anatomical and imaging-correlated studies emphasise a layered distal architecture, including a deep aponeurotic component with a wide coronoid footprint and a more compact superficial terminal tendon inserting anterolaterally; a retromolar extension has also been described (3, 9–11). Detailed anatomical work further supports internal organisation that can be described as a deep part or deep bundle within the TM, with distinct orientation and attachment features, and with separation of superficial and deep tendon layers described at the distal insertion (29, 30). Histochemical evidence supports regional fibre-type heterogeneity consistent with functional compartmentalisation within the TM (7, 31). This reference architecture provides the baseline morphological components used for the operational criteria of Type I (see Section 7.1). Study-level operational definitions and any reported *n*/*N* (%) are synthesised in Table 1.

### 4.2 Typical variations: number of bellies, tendinous extensions, and attachment differences

Across classical and contemporary sources, typical variability includes segmented architecture described as bi- and tripartite configurations or separable intramuscular compartments, layered or dual terminal tendons with retromolar extension, and accessory insertions to the anterior mandibular ramus or adjacent periosteum (4, 9–11, 27–29, 32). Differences in reported patterns are driven in part by heterogeneity in operational definitions, including what constitutes a discrete belly vs. intramuscular layering or compartmentalisation, and by variable denominators (specimens, heads, or sides) across studies (4, 9, 10). These recurring descriptions provide the morphological basis for the compartmentalisation component captured in Type II and the accessory attachment component captured in Type III. Study-level synthesis is provided in Table 1.

### 4.3 Additional fascial extensions and atypical attachments

The TM interfaces with the deep temporal fascia (DTF), superficial temporal fascia (STF), and temporoparietal fascia, and its distal and peritendinous morphology is shaped by these laminar envelopes and their regional continuity (2, 8). Variable fascial expansions and atypical tendinous attachments, including descriptions of attachments or extensions towards the anterior mandibular ramus, masseteric fascia, and the coronoid notch or retromolar region, have been reported and contribute to heterogeneity in nomenclature and reported morphology (4, 8–11, 14). Such expansions may blend with adjacent fascial planes rather than forming consistently separable structures, which partly explains discrepancies in terminology between authors and across modalities (4, 9). In the proposed framework, only substantive, traceable accessory bands or attachments that are operationally defined in primary studies are treated as Type III features, whereas thin fascial strands are considered part of normal peritendinous variability unless explicitly defined otherwise (11, 33). See Table 1 for study-level synthesis.

### 4.4 Rare, unnamed variants

Beyond recurring patterns, case reports and selected cadaveric observations describe uncommon configurations including accessory slips, atypical distal tendon configurations reported in isolated descriptions, unusual fibre orientations, and atypical attachments deviating from classical descriptions (9–11, 33). These observations broaden the morphological spectrum but generally lack harmonised definitions and consistent denominators, limiting comparability across studies and precluding robust prevalence estimation (4, 9). Such patterns should therefore be treated as hypothesis-generating observations until replicated with standardised, side-level reporting. Case-level evidence predominates and pooling is not attempted; see Table 1.

### 4.5 Historical and contemporary evidence (synthesis)

Classical treatises recognised the TM's wide origin, fan-like fibre arrangement, and occasional accessory slips (1, 26–28). Modern studies refine this view by documenting lamination and internal architecture, deep bundle concepts, dual terminal tendons with retromolar extension, segmented or compartmentalised patterns, and variable distal attachments using dissection, histology, innervation tracing, and imaging-correlated anatomy (3, 4, 9–11, 14, 29, 30, 32, 33). To minimise narrative redundancy and make heterogeneity in definitions and denominators explicit, we provide a comparative Evidence Map in Table 1 that links study-level operational definitions and reported frequencies to the morphological components used to operationalise the proposed Types I to IV. Studies focused primarily on developmental context or imaging technique, rather than morphological variant definitions, are summarised separately in the Supplementary Material. Imaging correlates and common diagnostic pitfalls are synthesised in Section 5, and procedural implications are discussed in Section 6.

## 5 Radiological features and diagnostic pitfalls

### 5.1 MRI and ultrasound imaging correlates and common interpretive pitfalls

Magnetic resonance imaging provides a consistent overview of TM morphology and the distal tendon complex. Imaging-correlated anatomical work describes a layered distal configuration, including a broad deep aponeurosis with a wide coronoid footprint and a more compact superficial terminal tendon inserting anterolaterally; a retromolar extension has also been described in anatomical series (10, 11). These descriptions provide the imaging context for identifying the distal components that are operationalised within the proposed framework.

A recurring interpretive issue is that thin tendinous laminae and small-calibre slips are vulnerable to partial-volume averaging and orientation-related artefacts, which can either exaggerate apparent separation or obscure small structures. These phenomena are general to musculoskeletal MRI, but they are particularly relevant here because distal tendon layers and any fine slips are close to the limits of routine spatial resolution. Consequently, apparent discontinuity or separation should be interpreted cautiously, particularly when it is seen in only a single plane or without confirmatory continuity of fibres across planes.

Accessory slips and bands can also create mass-like appearances if continuity with the parent temporalis muscle is not established. Features supporting a variant rather than pathology include muscle-like signal characteristics across sequences and anatomical continuity with temporalis fibres on multiplanar assessment. Published discussion of these interpretive pitfalls remains largely descriptive and case-based rather than supported by comparative diagnostic-accuracy studies, and the frequency of misinterpretation cannot be estimated reliably from current data (9, 34).

THE TM oedema and enhancement patterns represent a distinct diagnostic domain that should not be conflated with morphological variants. In giant cell arteritis, MRI may show temporalis oedema and enhancement with peri-arterial involvement of the deep temporal artery, and bilateral involvement has been reported in a subset of patients. In such scenarios, imaging appearances can overlap with infectious myositis or neoplastic infiltration, therefore interpretation requires correlation with systemic clinical features, inflammatory markers, and vascular assessment where clinically indicated (35).

Variation in TM thickness and cross-sectional appearance should also be interpreted in clinical context. Reduced bulk may reflect disuse associated with pain-related unloading or altered mastication, rather than neurogenic denervation. When neurogenic change is present, it is more commonly characterised by fluid-sensitive signal alterations in earlier phases and fatty infiltration on T1-weighted imaging in later phases, particularly when supported by electrophysiological findings. The available evidence does not support morphology-based thresholds that distinguish physiological variation from pathology without clinical correlation (36, 37).

Ultrasound can depict superficial architecture and dynamic behaviour of the TM, but it is operator dependent and susceptible to anisotropy, which may produce hypoechoic appearances that mimic tearing or focal abnormality. Dynamic assessment and systematic multiplanar evaluation can improve confidence that a focal band represents normal anatomy rather than pathology, particularly when continuity with parent fibres is demonstrable (9).

In the distal tendon and coronoid region, extraoral ultrasound windows may be constrained by acoustic shadowing from the zygomatic arch. Intraoral ultrasonography has been proposed as a complementary approach providing a direct window to the coronoid process and adjacent distal tendon, using the maxillary second molar region as a practical landmark for probe positioning in standardised conditions. This technique has been discussed primarily as an anatomical consideration for image-guided procedures in suspected temporal tendinitis rather than as a method to classify temporalis morphological variants at the population level (38).

Ultrasound elastography has been applied to masticatory muscles in temporomandibular disorder populations, where thickness and cross-sectional measures, and in some reports stiffness-related parameters, have been reported in association with symptom burden. However, protocol heterogeneity and limited reproducibility data constrain interpretability, and elastography should be regarded as adjunctive information rather than a standalone discriminator of morphology or pathology (37).

Computed tomography has limited soft-tissue contrast for detailed temporalis architecture, but it provides relevant osseous context and has been used in developmental imaging studies describing growth-related changes in temporalis morphology and spatial coverage. Such data are best treated as contextual information and should not be interpreted as definitional evidence for morphological variant categories (39).

This section synthesises imaging correlates and interpretive pitfalls relevant to recognising described anatomical components. It does not establish diagnostic-accuracy metrics or outcome-validated imaging criteria for the proposed types, because comparative studies stratified by temporalis morphological configuration are not yet available.

### 5.2 EMG and dynamic assessment

Electromyography assesses neuromuscular recruitment rather than gross morphology. In this context, a normal EMG together with reduced temporalis bulk on MRI or ultrasound is more consistent with disuse and under-recruitment than with denervation, whereas neurogenic loss is more often associated with characteristic MRI signal evolution, including early fluid-sensitive changes and later fatty infiltration on T1-weighted sequences, alongside electrophysiological abnormalities. These distinctions remain context dependent and require integration with symptoms, examination, and longitudinal change (36, 37).

Dynamic ultrasound can provide a functional adjunct by documenting contraction-associated thickening during clenching, which may support longitudinal interpretation when combined with clinical assessment. However, validated thresholds and outcome-linked criteria have not been established for temporalis morphological types, and dynamic measures should therefore be interpreted as supportive rather than determinative (37).

## 6 Surgical and clinical implications

The proposed Types I to IV are intended as a descriptive, side level framework to organise clinically relevant differences in TM architecture. The available evidence is derived predominantly from descriptive cadaveric studies and imaging correlated anatomy. Accordingly, the implications summarised below should be interpreted as hypothesis generating anatomical context designed to support consistent reporting and future prospective validation, rather than as outcome validated recommendations (3, 4, 9–11, 33).

### 6.1 Reconstructive and orthognathic relevance of temporalis variability

Temporalis based techniques are established options in craniofacial reconstruction and facial reanimation because the muscle is accessible and can be mobilised with preserved vascular supply when standard planes are respected (5, 8, 40). Interindividual variation in muscle volume, fascicular organisation, the distal tendon complex, and relationships to temporal fascial planes may influence mobilisation, reach, contour, and vector behaviour. These features are therefore relevant when planning temporalis transfer or lengthening temporalis myoplasty, particularly where layered distal architecture or accessory distal attachments may change the extent of release and the need for intraoperative retensioning (9–11, 14, 33, 41). Because comparative outcome studies stratified by TM morphological type are not currently available, type based statements in this section should be presented as an organising framework that formalises anatomical mapping rather than as a prescriptive operative algorithm (14, 41).

In orthognathic and mandibular ramus procedures, masticatory muscle forces contribute to postoperative mandibular biomechanics, and variation in distal attachments or partitioning may be relevant to local dissection planes and postoperative functional adaptation (42). However, direct evidence linking specific temporalis types to orthognathic outcomes is limited. Any type referenced statements should therefore remain explicitly interpretive and anatomical rather than outcome based (4, 9, 10).

### 6.2 Complications and interpretive errors related to unrecognised variability

Accessory slips, atypical distal attachments, or pronounced partitioning patterns may complicate identification of surgical planes and landmarks in temporal and craniofacial approaches and may increase operative complexity, particularly when the distal tendon region or retromolar anatomy is used as a functional landmark (4, 10, 11, 33). Injury to the frontal branch of the facial nerve remains a key iatrogenic concern when dissection deviates from established fascial planes, and careful attention to temporal fascial anatomy and recognised safety zones is central to operative safety in this region (8, 43). Temporal hollowing has been reported after temporal approaches and has been attributed to factors including muscle mobilisation, denervation, atrophy, and fixation technique, although the relative contribution of these factors varies across operative contexts and has not been evaluated in relation to the proposed temporalis types (9, 41).

On imaging, variant bundles or accessory bands can resemble focal pathology when assessed in limited planes or at inadequate resolution. In equivocal cases, interpretation should prioritise confirmation of fibre continuity and skeletal muscle consistent signal behaviour across orthogonal planes and sequences, with correlation to clinical context where appropriate (9, 34).

### 6.3 Temporomandibular disorders, myofascial pain, and intervention planning

Morphological variability, including differences in tendon layering, accessory slips, and regional hypertrophy or atrophy, may contribute to heterogeneity in symptom distribution and treatment response in temporomandibular disorders, although direct evidence remains limited (36, 37). Temporalis trigger points are commonly targeted in conservative management, and image guided localisation may improve anatomical precision in selected patients (37, 44). Because controlled studies comparing treatment response across the proposed morphological types are not available, any type referenced tailoring should be framed as structured anatomical mapping rather than as a validated treatment algorithm (4, 9, 10).

Distal temporalis tendon pain and temporal tendinitis may mimic or coexist with temporomandibular disorder symptomatology and can be under recognised clinically. Intraoral ultrasound has been described as a complementary technique to visualise the coronoid region and adjacent distal tendon anatomy, supporting anatomically informed injection planning when extraoral windows are limited (38). For botulinum toxin and other injection based interventions, ultrasound may assist in documenting layer depth and potential accessory bands to support anatomically consistent targeting. However, no controlled trials have systematically tailored injection patterns to temporalis morphological type and compared clinical outcomes (37).

### 6.4 Oncologic context and temporalis morphometry

In head and neck oncology, temporalis muscle thickness has been used as an objective morphometric marker associated with systemic status and prognosis in patient cohorts, illustrating the potential clinical relevance of temporalis measurements beyond local anatomy (37). Within the scope of the present review, any extension of the proposed morphological framework into oncologic planning or follow up should be regarded as hypothesis generating, as temporalis specific, type stratified validation linking morphology to treatment constraints or functional outcomes has not yet been performer (45, 46).

## 7 Classification: definitions and assignment

Scope. Assignment is performed on a side-level (right/left) based on intra-operative anatomy or MRI/US. Purely functional alterations (oedema, atrophy, fibrosis) do not determine type; the defining criteria are fascicular architecture and the configuration of the distal tendon complex.

In contrast to prior descriptions that have focused separately on intramuscular layering (4, 9) or distal tendon and retromolar variation (10, 11), the scheme below integrates belly architecture, tendon configuration and accessory slips into a single side-level framework with explicit imaging and surgical correlates.

### 7.1 Definitions of morphological types (types I–IV)

Type I — classical fan-shaped (monobellied).

Single muscle mass without a discrete second belly; distal complex with a deep aponeurosis and a superficial terminal tendon inserting on the coronoid process; a thin retromolar extension may be present; no independent accessory attachment. *Imaging correlates*: continuous fibres in ≥2 planes; muscle-like signal; no separate slip behaving as a mass.

Type II — bifid/bipartite bellies.

Two partially separated bellies (tendinous septum/cleft that is visible on ≥2 contiguous MRI slices or as a reproducible separation plane on dynamic ultrasound) converging distally; at least partial independence of terminal bands (one may be volumetrically dominant). Imaging correlates: two fibre bundles with stable continuity to the distal insertion on orthogonal MRI or dynamic US; the separation should be demonstrable in more than one plane to minimise misclassification from partial-volume or anisotropy artefacts.

Type III — accessory slips/tendinous extensions.

Presence of an additional slip/band continuous with the temporalis muscle that inserts beyond the usual footprint (e.g., anterior border of the mandibular ramus, masseteric fascia, retromolar triangle/notch) or a retromolar band of non-trivial calibre, i.e., clearly thicker and more muscle-like than a thin fascial strand and traceable as a discrete fibre bundle. Imaging correlates: muscle-like signal, contraction on US, typically avascular on Doppler; continuity confirmed in ≥2 planes rather than inferred from a single oblique slice.

Type IV — complex/mixed.

Co-occurrence of features of Types II/III or an atypical multilaminar pattern not fitting I–III. *Imaging correlates*: multiple bands/laminae; oblique planes are often required, and intra-operative confirmation may be needed.

Adjudication rules. Assign by architecture (not by size or signal change alone). No absolute numerical cut-offs for thickness or cross-sectional area are specified, because existing datasets do not support evidence-based thresholds; pattern and continuity should take precedence over diameter. If criteria overlap, assign the highest applicable type. Report laterality (unilateral/bilateral) and symmetry. Table 1 links the supporting literature to the morphological components operationalised in Types I–IV, and Table 2 summarises the definitions, imaging correlates, and study-level evidence for rapid reference.

**Table 2:** Classification quick reference for the temporalis muscle (types I to IV).

Type	Defining anatomical features	Imaging correlates	Clinical implications	Evidence base
I	Single belly. Distal complex characterised by deep aponeurosis and superficial terminal tendon inserting on the coronoid process. A thin retromolar extension may be present. No independent accessory attachment.	Continuous fibres confirmed in at least two planes. Signal characteristics consistent with skeletal muscle across sequences. No discrete accessory slip with mass-like behaviour.	Represents the reference architecture against which additional partitions or accessory attachments are interpreted. Relevant to baseline expectations in flap harvest and myoplasty planning.	Supported by cadaveric and imaging-correlated anatomy describing layered architecture and the distal tendon complex (3, 9, 10).
II	Two partially separated bellies or clearly separable intramuscular compartments, with a persistent septum or cleavage plane appreciable on repeat assessment, converging distally. Partial independence of terminal bands may be present.	Two fibre bundles or compartments that can be tracked to the distal insertion on orthogonal MRI planes or demonstrated as a reproducible separation plane on dynamic ultrasound.	May influence side-specific force vectors and procedural planning where differential detachment, tensioning, or preservation of components is relevant. Laterality and symmetry should be documented.	Supported by anatomical series describing intrinsic partitioning or deep bundle(s) of the temporalis and their relations/innervation, although prevalence cannot be estimated reliably owing to heterogeneous denominators and definitions (4, 29, 30, 32).
III	Accessory slip or band continuous with temporalis inserting beyond the usual footprint, for example onto the anterior mandibular ramus or retromolar region, or other substantive accessory attachments near the insertion.	Signal characteristics consistent with skeletal muscle. Contraction on ultrasound when assessed dynamically. Continuity should be confirmed in at least two planes to reduce artefact-related misclassification.	May modify distal dissection planes and the configuration of the distal tendon region. Relevant when accessory attachments are present near operative corridors or injection targets, and when distinguishing normal slips from pathology on imaging.	Supported by cadaveric series documenting retromolar extension and substantive accessory attachments near the insertion, although large-scale prevalence data are not available (10, 11, 29, 33).
IV	Composite pattern with combined occurrence of features consistent with Types II and III, or atypical multilaminar configuration not fitting Types I to III.	Multiple bands or laminae that may require oblique planes and, in equivocal cases, intraoperative confirmation.	Represents a higher complexity configuration that may require individualised mapping of partitions and accessory attachments. Should be reported explicitly as mixed and side-specific.	Conceptual composite category introduced to capture overlap between separable belly and accessory attachment components. Not supported by dedicated quantitative series and should be interpreted as hypothesis generating (4).

The evidence base reflects study-level anatomical and imaging-correlated descriptions mapped to the components of each type (Table 1) and should not be interpreted as population prevalence estimates.

A quick-reference summary of defining features, imaging correlates and clinical implications is provided in Table 2.

### 7.2 Algorithm 1 — MRI/US-guided assignment (side-level)

Algorithm 1 outlines a concise MRI/US decision pathway for side-level assignment. The pathway assumes confirmation of fibre/tendon continuity in at least two planes or intra-operatively.

Algorithm 1. **MRI/US-guided assignment for temporalis muscle classification (side-level). It is intended as a pragmatic, easy-to-apply guide rather than statistical proof of discrete morphological clusters.**

Scope

Side-level assignment based on intra-operative anatomy or MRI/ultrasound. Types are defined by fascicular architecture and the configuration of the distal tendon complex; functional alterations alone (oedema, atrophy, fibrosis) do not determine type.

Inputs

MRI: T1 axial and coronal plus oblique sagittal along the ramus/coronoid; T2-FS/PD-FS; consider diffusion-weighted imaging (DWI) if required.
Ultrasound: high-frequency linear probe ± Doppler; dynamic manoeuvres, consider intraoral US for distal tendon/coronoid assessment when extraoral windows are limited (38).

Output

Type I/Type II/Type III/Type IV

Quality gate

Confirm fibre/tendon continuity in ≥2 planes or intra-operatively before assignment.
If insufficient, add oblique planes and/or DWI, or perform dynamic ultrasound, then re-assess.

Decision steps

Two discrete bellies separated by a tendinous septum, both converging distally — assign Type II; otherwise proceed to step 2.
Accessory slip or extra attachment beyond the usual footprint (e.g., anterior mandibular ramus, masseteric fascia, retromolar triangle/notch) of non-trivial calibre, with continuity to the temporalis in ≥2 planes — assign Type III; otherwise proceed to step 3.
Composite/atypical multilaminar pattern (overlapping features of Types II and III) — assign Type IV; otherwise assign Type I.

Tie-breaker

If criteria of Types II and III co-exist, assign Type IV.

Equivocal imaging

When classification remains inconclusive, verify intra-operatively and update the assignment *post hoc*.

Documentation

Record type, side, and symmetry (bilateral/asymmetric).

For a concise side-level summary, see Table 2.

### 7.3 Evidence supporting the types

No pooled prevalence is presented because operational definitions and denominators vary substantially across studies (specimens vs. heads vs. sides), and side-level reporting is inconsistent. Accordingly, the evidentiary basis for the proposed scheme is presented as a study-level synthesis that links extractable definitions and reported frequencies to the morphological components operationalised in Types I–IV (Table 1), with a concise summary of definitions, imaging correlates, and supporting sources in Table 2.

Type I is supported as the reference architecture by convergent cadaveric and imaging-correlated descriptions of layered intramuscular organisation and the distal tendon complex. Studies describing lamination within a single temporalis unit, rather than a separate accessory muscle, provide the core anatomical basis for the Type I criteria (3, 9). The configuration of the distal tendon complex, including a broad deep aponeurosis and a more compact superficial terminal tendon inserting on the coronoid process, is consistently described in dedicated anatomical series (10). Reports describing a deep part or deep bundle of the temporalis and its orientation further support interpretation of such internal architecture as compartmentalisation within the temporalis rather than a distinct entity, aligning with the Type I reference pattern (29, 30).

Type II is supported by studies describing reproducible partitioning or separable compartments within the temporalis muscle that can be distinguished by fibre organisation and regional anatomy, although terminology and thresholds for declaring a discrete belly vary between authors. Cadaveric and imaging-correlated work describing intrinsic parts of the temporalis, including separable components or a deep bundle, supports the compartmentalisation component that underpins the operational Type II definition (4, 29, 30, 32). Because these datasets apply different labels and denominators, they support the existence of partitioning but do not enable reliable prevalence estimates for a specific two-belly configuration as defined here.

Type III is supported by cadaveric series documenting substantive accessory distal attachments and extensions beyond the usual footprint, particularly in the retromolar and anterior ramus region. The distal temporalis tendon extension to the retromolar triangle and adjacent anterior mandibular ramus has been operationalised as a consistent anatomical feature in a dedicated dissection series, supporting the accessory-attachment component of Type III (11). Additional cadaveric work mapping accessory attachments near the main insertion and relating these to surgically relevant anatomy further supports the existence of substantive, traceable accessory bands (33). Detailed morphometric descriptions of the coronoid footprint and the dual terminal tendon arrangement provide additional context for distinguishing thin retromolar components within reference architecture from more substantive accessory attachments that meet Type III criteria (10).

Type IV is an explicitly hypothesis-generating composite category introduced to capture overlap between compartmentalisation features and substantive accessory attachments, as well as atypical multilaminar configurations that do not fit Types I–III. It is not underpinned by dedicated quantitative series that prospectively define and count complex mixed patterns using harmonised criteria; rather, it is derived from synthesis across heterogeneous descriptive reports in which overlapping features are variably labelled or discussed (4). Type IV should therefore be interpreted as a pragmatic organisational label to support consistent reporting in complex cases, pending prospective validation with standardised, side-level definitions.

Across all types, the current evidence base is dominated by descriptive cadaveric studies and imaging-correlated anatomical reports rather than population-based cohorts or outcome-linked registries (3, 4, 9–11, 29, 30, 33). The proposed scheme should therefore be interpreted as an expert synthesis and a structured, operational framework to organise existing observations and facilitate prospective, standardised validation rather than as a statistically validated taxonomy or prevalence model.

## 8 Biomechanics and function of the temporalis muscle

The TM generates mandibular elevation, with posterior fibres contributing to retrusion and stabilisation during complex jaw movements. Vector behaviour is determined by fascicular architecture and the distal tendon complex, including intramuscular lamination and the insertional footprint on the coronoid process (3). Interindividual variation in compartmentalisation and distal attachments can plausibly alter moment arms and side-to-side recruitment symmetry, thereby influencing bite kinematics and load distribution, although type-stratified biomechanical validation remains limited (31). In the present framework, side-level assignment by Algorithm 1 is intended to provide a structured way to report expected vector behaviour, rather than to claim outcome-validated functional differences.

Type-linked functional expectations should be stated cautiously and framed as anatomical inference from morphology. For Type I, a single fan-shaped belly with layered distal tendon components provides the reference configuration, with a thin retromolar extension (when present) typically considered part of baseline architecture rather than a distinct auxiliary vector (3, 10). For Type II, persistent compartmentalisation or a reproducible cleavage plane implies partial mechanical independence of regions, which may bias elevation or retrusion vectors on the affected side depending on the dominant component, but this remains hypothesis-generating without prospective functional correlation (4). For Type III, a substantive accessory slip or distal attachment beyond the usual footprint may add an auxiliary anterior or lateral vector component and may alter local tension distribution at the distal complex (10, 11, 33). For Type IV, overlapping compartmentalisation and accessory attachment features imply greater vector complexity and reinforce the rationale for deliberate side-level mapping and explicit reporting rather than assumptions based on a single reference pattern (4).

Electromyography reflects recruitment rather than structure; therefore, normal EMG in the context of reduced TM bulk on imaging is more consistent with underuse or disuse than denervation, whereas neurogenic change is more often associated with characteristic imaging evolution and electrophysiological abnormality (36). In temporomandibular disorder and myofascial pain contexts, local architecture may matter because compartment boundaries, tendon–muscle junctions, and slip interfaces can act as mechanically relevant regions where trigger points may be clinically targeted; in practice, mapping should focus on reproducible anatomy rather than assuming uniform distribution within the muscle (9, 10).

## 9 Immunohistochemistry and molecular aspects of the temporalis muscle

Histochemical studies demonstrate that the TM contains a mosaic of fibre types, with regional heterogeneity across superficial vs. deep and anterior vs. posterior portions (7, 31). These regional differences are consistent with functional compartmentalisation, in which anterior and superficial regions tend to support rapid force generation while deeper or posterior regions may support more tonic components of mandibular control (7, 31). Such heterogeneity provides a biological rationale for why partitioning patterns and layered architecture described anatomically may be functionally meaningful, while also reinforcing that “type” assignment is morphological and should not be equated with a specific fibre-type profile without direct evidence.

Clinically oriented interpretations should remain conservative. Regional fibre-type differences may contribute to variability in fatigue behaviour and pain sensitivity across the TM, and junctional regions where laminae, septa, or accessory slips converge on the distal complex may represent mechanically relevant sites for symptom generation or procedural targeting (9, 10). Where interventions such as dry needling or injection-based therapies are used, the implication of the present framework is that targeting may benefit from side-level anatomical localisation of the relevant belly, compartment, or accessory band when such features are present, rather than uniform dosing or uniform sampling across the muscle (37, 44). At present, however, there are no controlled studies linking the proposed Types I–IV to specific immunohistochemical signatures or differential treatment response, and such statements should be framed as structured anatomical reasoning rather than validated biological stratification (7, 31).

## 10 Advanced imaging techniques in temporalis muscle assessment

Standard clinical MRI and high-frequency ultrasound remain the reference modalities for evaluating temporalis morphology and dynamic behaviour and underpin the side-level assignment rules described in Algorithm 1. Their principal limitations in this context are resolution- and orientation-related artefacts for MRI and operator dependence and anisotropy for ultrasound, which are particularly relevant when assessing thin tendon laminae or small-calibre slips (9).

Advanced modalities may add value in selected scenarios, but temporalis-specific validation is uneven and should be described as adjunctive rather than definitive. Ultra-high-field MRI (for example 7T) can improve delineation of small structures and tendon laminae and may help resolve equivocal patterns when standard imaging is inconclusive, although availability and susceptibility artefacts limit routine use (14). Ultrasound elastography has been applied to masticatory muscles in temporomandibular disorder populations and may provide adjunctive information on stiffness-related parameters and longitudinal change, but protocol heterogeneity and limited reproducibility data constrain standalone interpretability (37). In oncologic contexts, temporalis thickness on cross-sectional imaging has been used as a morphometric marker associated with systemic status and prognosis, illustrating that temporalis measurements can carry clinical information beyond local anatomy, albeit not specifically linked to Types I–IV (45, 46). A structured summary of incremental value, limitations, and typical indications across advanced modalities is provided in Table 3, and should be interpreted as a pragmatic overview rather than an evidence-graded recommendation set.

**Table 3:** Advanced imaging of the temporalis muscle: incremental value, limitations and clinical indications.

Modality	Adds beyond standard MRI/US	Main limitations/pitfalls	Typical clinical indications
7T MRI (ultra-high field)	Resolves small accessory slips and distal tendon laminae; refines Type III/IV assignment; sharper anatomical margins	Limited availability; susceptibility artefacts; higher cost	Equivocal patterns on standard imaging; detailed pre-operative mapping for vector planning
DTI/tractography	Fibre-direction mapping and anisotropy; aligns with belly/septal layout; depicts intramuscular architecture	Angle/motion sensitivity; protocol heterogeneity; expertise required	Complex mixed (Type IV) where fibre course informs surgical or rehabilitation strategy
Quantitative fat-fraction (Dixon/IDEAL)	Objective fat content to differentiate chronic denervation-related fatty change from disuse	Partial-volume effects; availability; need for standardisation	Ambiguous weakness; supports prognosis and tailoring of dosing/rehabilitation intensity
MR elastography	Whole-muscle stiffness estimate with reduced operator dependence compared with ultrasound	Limited availability; motion susceptibility; validation evolving	Selected cases where global stiffness measurement may guide load progression
US elastography (shear-wave/strain)	Focal stiffness at slip-tendon junctions; bedside and repeatable; aligns with trigger-point mapping	Operator/angle dependence; preload and jaw-position artefacts	Longitudinal tracking after botulinum toxin or myoplasty; focal pain mapping
PET/MRI hybrid	Combines metabolic and anatomical information (inflammation/neoplasia/overuse)	Very high cost; limited accessibility	Complex diagnostic work-ups only
Standard MRI/ultrasound (reference)	Baseline morphology and dynamic assessment; supports type assignment per Algorithm 1	MRI: magic-angle and partial-volume effects; US: operator dependence	Routine assessment; triage for any advanced add-on imaging

## 11 Future directions and research gaps

### 11.1 Lack of standardized population-based studies

Current literature lacks large-scale, population-based studies assessing the morphological variability of the TM. Most studies are limited by small sample sizes or cadaveric observations that may not represent broader population diversity (47). Establishing comprehensive normative data requires well-designed epidemiological investigations across different demographics (age, sex, ethnicity).

### 11.2 Need for correlation between morphology and function

While anatomical variability is described, there is insufficient data correlating morphology with functional outcomes. Studies integrating electromyography and biomechanical analysis with morphological assessment remain scarce but are essential to understand the clinical significance of variants, especially in TMD (37, 44).

### 11.3 Role of immunohistochemistry and advanced imaging techniques

Emerging techniques such as immunohistochemistry and advanced imaging (e.g., diffusion tensor imaging, ultrasound elastography) have begun to reveal microstructural muscle features and functional properties that standard imaging cannot capture. These methods can identify fiber type distribution, innervation, and biomechanical properties relevant to both normal variation and pathology (37).

### 11.4 Proposal for standardization of terminology and classification

The heterogeneity in terminology and classification systems for TM morphology hampers comparability across studies and clinical practice. Recent calls for unified classification frameworks emphasize the need to integrate gross anatomy, histology, and imaging data into consistent, reproducible schemes (47).

### 11.5 Validation and clinical translation

Prospective, multicentre studies using harmonised operational definitions and side-level reporting are needed to establish robust prevalence estimates. Standardised MRI protocols (including agreed planes/sequences) and pre-registered cadaveric protocols would reduce bias and enable meta-analysis. Clinical registries linking variant morphology to surgical outcomes should validate the proposed classification. These priorities directly address the sources of heterogeneity summarised in Table 1 and are expected to convert study-level evidence into pooled, clinically actionable estimates.

## 12 Limitations

Despite extensive investigation into the morphological variability of the TM, several limitations constrain the current body of knowledge and its clinical translation.

Firstly, many anatomical studies rely on relatively small sample sizes, often based on cadaveric dissections from limited demographic groups. Such samples may not adequately represent population-wide variability, particularly across different ethnicities, ages, and sexes. This limits the generalizability of findings and the establishment of comprehensive normative data.

Secondly, imaging studies face inherent technical constraints. While MRI and ultrasound provide valuable insights into muscle morphology and function, their resolution and operator dependency introduce variability in measurements (37). The lack of standardized imaging protocols for TM assessment further complicates cross-study comparisons.

Thirdly, most functional correlations between morphology and muscle performance are derived from indirect methods such as electromyography (EMG) or clinical assessments, which do not fully capture complex neuromuscular interactions. Direct *in vivo* studies combining high-resolution imaging with functional metrics remain scarce.

Additionally, histological and immunohistochemical analyses, essential for understanding muscle fiber composition and innervation patterns, are limited by the invasive nature of sample collection and ethical considerations. Consequently, data on cellular-level variability in the TM is sparse.

Finally, the diversity in classification systems and inconsistent terminology across studies impedes the integration of anatomical, imaging, and clinical data into unified frameworks, reducing translational impact.

Addressing these limitations requires larger, multiethnic cohort studies employing standardized imaging and functional assessment protocols, alongside advanced histological techniques. Establishing consensus nomenclature and classification standards will further enhance research comparability and clinical applicability.

Across cadaveric and imaging series, definitions of “variant” and denominators (specimens vs. sides vs. heads) are inconsistent, with limited blinding and sparse inter-rater data. Frequencies are often unreported, and case reports inflate the visibility of rare patterns. Given this heterogeneity, pooling was not attempted; instead, we provide a study-level synthesis (Table 1). In this context, the four-type classification proposed here should be viewed as a pragmatic, hypothesis-generating organisational tool rather than a statistically validated taxonomy, pending confirmation in prospective, standardised datasets.

## 13 Conclusion

The temporalis muscle (TM) exhibits clinically meaningful morphological variability that influences both imaging interpretation and operative planning. In this review, we synthesised heterogeneous cadaveric and imaging-correlated descriptions of layered intramuscular organisation, distal tendon architecture, retromolar extension, and accessory attachments, and aligned study-level definitions and frequencies using an evidence map (Table 1). On this basis, we propose a pragmatic, side-level, four-type classification with operational criteria and an MRI/ultrasound assignment pathway intended to support consistent reporting across disciplines (Table 3).

Because the underlying evidence is dominated by descriptive series with variable denominators and non-standardised definitions, the proposed types should be regarded as hypothesis-generating rather than prevalence-based categories. Prospective validation will require harmonised criteria, routine side-level reporting, and reproducible imaging protocols, together with linkage to clinically relevant outcomes in reconstructive, orthognathic, pain-therapy, and oncologic contexts. Establishing such datasets will determine whether type-stratified approaches can improve diagnostic precision, refine vector planning, and reduce complications.

## References

[1] Bergman RA. Illustrated Encyclopedia of Human Anatomic Variation: Opera. Iowa City, IA: Virtual Hospital (1996).

[2] Standring S. Gray’s Anatomy: The Anatomical Basis of Clinical Practice. Philadelphia, New York: Elsevier (Churchill Livingstone) (2016).

[3] Geers C Nyssen-Behets C Cosnard G Lengelé B. The deep belly of the temporalis muscle: an anatomical, histological and MRI study. Surg Radiol Anat. (2005) 27:184–91. 10.1007/s00276-004-0306-3 15821860

[4] Sedlmayr JC Kirsch CF Wisco JJ. The human temporalis muscle: superficial, deep, and zygomatic parts comprise one structural unit. Clin Anat. (2009) 22:655–64. 10.1002/ca.20837 19637294

[5] Marwan H Hardman C. The use of split temporalis muscle flap in maxillofacial reconstruction. Craniomaxillofac Trauma Reconstr Open. (2021) 6:247275122110308. 10.1177/24727512211030836

[6] Brennan T Tham TM Costantino P. The temporalis muscle flap for palate reconstruction: case series and review of the literature. Int Arch Otorhinolaryngol. (2017) 21:259–64. 10.1055/s-0037-1598653 28680495 PMC5495588

[7] Eriksson PO Thornell LE. Histochemical and morphological muscle-fibre characteristics of the human masseter, the medial pterygoid and the temporal muscles. Arch Oral Biol. (1983) 28:781–95. 10.1016/0003-9969(83)90034-1 6227313

[8] Labbé D Huault M. Lengthening temporalis myoplasty and lip reanimation. Plast Reconstr Surg. (2000) 105:1289–97; discussion 1298. 10.1097/00006534-200004000-00005 10744217

[9] Lee JY Kim JN Kim SH Choi HG Hu KS Kim HJ Anatomical verification and designation of the superficial layer of the temporalis muscle. Clin Anat. (2012) 25:176–81. 10.1002/ca.21212 21739477

[10] Yu SK Kim TH Yang KY Bae CJ Kim HJ. Morphology of the temporalis muscle focusing on the tendinous attachment onto the coronoid process. Anat Cell Biol. (2021) 54:308–14. 10.5115/acb.21.074 34353976 PMC8493017

[11] Benninger B Lee B-I. Clinical importance of morphology and nomenclature of distal attachment of temporalis tendon. J Oral Maxillofac Surg. (2012) 70:557–61. 10.1016/j.joms.2011.02.047 21549487

[12] Landfald IC Olewnik Ł. An interdisciplinary review of the zygomaticus muscles: anatomical variability, imaging modalities, and clinical implications. J Clin Med. (2025) 14:4110. 10.3390/jcm14124110 40565856 PMC12194095

[13] Landfald IC Olewnik Ł. Are we underestimating zygomaticus variability in midface surgery? J Clin Med. (2025) 14:7311. 10.3390/jcm14207311 41156181 PMC12565131

[14] Landfald IC Vazquez T Okoń A Olewnik Ł. Temporalis muscle flap in craniofacial reconstruction: anatomy, techniques, outcomes, and innovations. Front Surg. (2025) 12:1678935. 10.3389/fsurg.2025.1678935 41141694 PMC12547924

[15] Bardeen CR Lewis WH. Development of the limbs, body-wall and back in man. Am J Anatomy. (1901) 1:1–35. 10.1002/aja.1000010102

[16] Bardeen C. Development and variation of the nerves and the musculature of the inferior extremity and of the neighboring regions of the trunk in man. Am J Anatomy. (1906) 6:259–390. 10.1002/aja.1000060108

[17] Couly GF Coltey PM Le Douarin NM. The developmental fate of the cephalic mesoderm in quail-chick chimeras. Development. (1992) 114:1–15. 10.1242/dev.114.1.1 1576952

[18] Rinon A Lazar S Marshall H Büchmann-Møller S Neufeld A Elhanany-Tamir H Cranial neural crest cells regulate head muscle patterning and differentiation during vertebrate embryogenesis. Development. (2007) 134:3065–75. 10.1242/dev.002501 17652354

[19] Burroughs-Garcia J Sittaramane V Chandrasekhar A Waters ST. Evolutionarily conserved function of Gbx2 in anterior hindbrain development. Dev Dyn. (2011) 240:828–38. 10.1002/dvdy.22589 21360792

[20] Buckingham M Rigby PW. Gene regulatory networks and transcriptional mechanisms that control myogenesis. Dev Cell. (2014) 28:225–38. 10.1016/j.devcel.2013.12.020 24525185

[21] Noden DM Trainor PA. Relations and interactions between cranial mesoderm and neural crest populations. J Anat. (2005) 207:575–601. 10.1111/j.1469-7580.2005.00473.x 16313393 PMC1571569

[22] Moss ML. The functional matrix hypothesis revisited. 1. The role of mechanotransduction. Am J Orthod Dentofacial Orthop. (1997) 112:8–11. 10.1016/S0889-5406(97)70267-1 9228835

[23] Chung PC Lin KK Song HS Ku WC Huang SC Sun CC. Alport syndrome with recurrent herpes simplex virus keratitis. Cornea. (2007) 26:1279–81. 10.1097/ICO.0b013e31814da529 18043194

[24] Subramanian A Schilling TF. Tendon development and musculoskeletal assembly: emerging roles for the extracellular matrix. Development. (2015) 142:4191–204. 10.1242/dev.114777 26672092 PMC4689213

[25] Nickel JC Iwasaki LR Gonzalez YM Gallo LM Yao H. Mechanobehavior and ontogenesis of the temporomandibular joint. J Dent Res. (2018) 97:1185–92. 10.1177/0022034518786469 30004817 PMC6151909

[26] Macalister A. Additional observations on muscular anomalies in human anatomy (third series), with a catalogue of the principal muscular variations hitherto published, in: *Trans Roy Irish Acad Sci*. (1875).

[27] Testut L. (1884). *Les anomalies musculaires chez l’homme expliquées par l’anatomie comparée et leur importance en anthropologie**.*

[28] Ledouble A. Traité des Variations du Système Musculaire de L’Homme et de Leur Signification au Point de Vue de L’Anthropologie Zoologique. Paris: Schleicher Frères (1897).

[29] Kageyama M Itoh I. Orientation of the deep part of the human temporal muscle and morphological study of the infratemporal crest. Japanese J Oral Biol. (2003) 45:397–406. 10.2330/joralbiosci1965.45.397

[30] Palomari E Picosse L Tobo M Isayama R Cunha M. Sphenomandibular muscle or deep bundle of temporal muscle? Int J Morphol. (2013) 31:1158–61. 10.4067/S0717-95022013000400002

[31] Korfage JA Koolstra JH Langenbach GE Van Eijden TM. Fiber-type composition of the human jaw muscles–(part 2) role of hybrid fibers and factors responsible for inter-individual variation. J Dent Res. (2005) 84:784–93. 10.1177/154405910508400902 16109985

[32] Shimokawa T Akita K Soma K Sato T. Innervation analysis of the small muscle bundles attached to the temporalis: truly new muscles or merely derivatives of the temporalis? Surg Radiol Anat. (1998) 20:329–34. 10.1007/BF01630615 9894312

[33] Prasidha I Singla A Rawther T Ngo Q. The temporalis muscle and its relationship to the accessory attachments and the main pedicle-a cadaveric study. J Plast Reconstr Aesthet Surg. (2020) 73:1122–9. 10.1016/j.bjps.2020.01.020 32139338

[34] Hassanein AG. Continuous validity of temporalis muscle flap in reconstruction of postablative palatomaxillary defects. J Craniofac Surg. (2017) 28:e130–7. 10.1097/SCS.0000000000003323 28033186

[35] Veldhoen S Klink T Geiger J Vaith P Glaser C Ness T MRI Displays involvement of the temporalis muscle and the deep temporal artery in patients with giant cell arteritis. Eur Radiol. (2014) 24:2971–9. 10.1007/s00330-014-3255-1 24895039

[36] Palinkas M Bataglion C De Luca Canto G Machado Camolezi N Theodoro GT Siéssere S Impact of sleep bruxism on masseter and temporalis muscles and bite force. Cranio. (2016) 34:309–15. 10.1080/08869634.2015.1106811 27077268

[37] Lee Y-H Bae H Chun Y-H Lee J-W Kim H-J. Ultrasonographic examination of masticatory muscles in patients with TMJ arthralgia and headache attributed to temporomandibular disorders. Sci Rep. (2024) 14:8967. 10.1038/s41598-024-59316-9 38637633 PMC11026518

[38] Kim SB Bae H Lee KW Hu KS Abe S Kim HJ. Anatomical consideration of ultrasonography-guided intraoral injection for temporal tendinitis. Clin Anat. (2024) 37:628–34. 10.1002/ca.24130 38146193

[39] Moltoni G D'arco F Rossi-Espagnet MC James G Hayward R. Observations on the growth of temporalis muscle: a 3D CT imaging study. J Anat. (2021) 238:1218–24. 10.1111/joa.13370 33280101 PMC8053578

[40] Bradley P Brockbank J. The temporalis muscle flap in oral reconstruction. A cadaveric, animal and clinical study. J Maxillofac Surg. (1981) 9:139–45. 10.1016/S0301-0503(81)80034-3 6944416

[41] Spetzler RF Lee KS. Reconstruction of the temporalis muscle for the pterional craniotomy. Technical note. J Neurosurg. (1990) 73:636–7. 10.3171/jns.1990.73.4.0636 2398396

[42] Xiangdong QI Limin MA Shizhen Z. The influence of the closing and opening muscle groups of jaw condyle biomechanics after mandible bilateral sagittal split ramus osteotomy. J Craniomaxillofac Surg. (2012) 40:e159–164. 10.1016/j.jcms.2011.07.024 21907586

[43] De Bonnecaze G Chaput B Filleron T Al Hawat A Vergez S Chaynes P. The frontal branch of the facial nerve: can we define a safety zone? Surg Radiol Anat. (2015) 37:499–506. 10.1007/s00276-014-1386-3 25342224

[44] Blasco-Bonora PM Martín-Pintado-Zugasti A. Effects of myofascial trigger point dry needling in patients with sleep bruxism and temporomandibular disorders: a prospective case series. Acupunct Med. (2017) 35:69–74. 10.1136/acupmed-2016-011102 27697769

[45] Lee B Bae YJ Jeong W-J Kim H Choi BS Kim JH. Temporalis muscle thickness as an indicator of sarcopenia predicts progression-free survival in head and neck squamous cell carcinoma. Sci Rep. (2021) 11:19717. 10.1038/s41598-021-99201-3 34611230 PMC8492642

[46] Lee HJ Jung SJ Kim ST Kim HJ. Ultrasonographic considerations for safe and efficient botulinum neurotoxin injection in masseteric hypertrophy. Toxins. (2021) 13:28. 10.3390/toxins13010028 33406757 PMC7824038

[47] Kim MJ Kim HB Jeong WS Choi JW Kim YK Oh TS. Comparative study of 2 different innervation techniques in facial reanimation: cross-face nerve graft-innervated versus double-innervated free gracilis muscle transfer. Ann Plast Surg. (2020) 84:188–95. 10.1097/SAP.0000000000002034 31688275

