# Supplementary material for: Morphological classification of the temporalis muscle: anatomical, radiological, and surgical perspectives
Source: Front Surg. 2026 Mar 3;13:1704668. doi: 10.3389/fsurg.2026.1704668 (PMC13040348; doi:10.3389/fsurg.2026.1704668)
Supplement: Supplementary file 1 [file Table1.docx]

**Supplementary Table S1.**

*Coverage: inception to 11 November 2025. Limits: humans; English language. No study-design or date filters applied at database level.*

| **Database (platform)** | **Core concept blocks (Boolean logic)** | **Full executable search string** | **Notes and filters** |
| --- | --- | --- | --- |
| MEDLINE (PubMed) | Block A (temporalis muscle): ("Temporalis Muscle"[Mesh] OR temporalis[tiab] OR "temporal muscle"[tiab]) AND Block B (structure and variation): (tendon[tiab] OR aponeurosis[tiab] OR fascia[tiab] OR "deep temporal fascia"[tiab] OR "superficial temporal fascia"[tiab] OR variation[tiab] OR variants[tiab] OR variability[tiab] OR morphology[tiab] OR anatomy[tiab]) AND Block C (imaging and clinical relevance): ("Magnetic Resonance Imaging"[Mesh] OR "magnetic resonance imaging"[tiab] OR "magnetic resonance"[tiab] OR ultrasonography[Mesh] OR ultrasonography[tiab] OR ultrasound[tiab] OR sonography[tiab] OR orthognathic[tiab] OR reconstructive[tiab] OR surgery[tiab] OR surgical[tiab]) | (("Temporalis Muscle"[Mesh] OR temporalis[tiab] OR "temporal muscle"[tiab]) AND (tendon[tiab] OR aponeurosis[tiab] OR fascia[tiab] OR "deep temporal fascia"[tiab] OR "superficial temporal fascia"[tiab] OR variation[tiab] OR variants[tiab] OR variability[tiab] OR morphology[tiab] OR anatomy[tiab]) AND ("Magnetic Resonance Imaging"[Mesh] OR "magnetic resonance imaging"[tiab] OR "magnetic resonance"[tiab] OR ultrasonography[Mesh] OR ultrasonography[tiab] OR ultrasound[tiab] OR sonography[tiab] OR orthognathic[tiab] OR reconstructive[tiab] OR surgery[tiab] OR surgical[tiab])) AND (english[lang]) | Apply Humans limit in PubMed if needed. Do not use truncation or wildcards; full terms only. |
| Embase (Elsevier) | Block A (temporalis muscle): ('temporalis muscle'/exp OR temporalis:ti,ab OR 'temporal muscle':ti,ab) AND Block B (structure and variation): (tendon:ti,ab OR aponeurosis:ti,ab OR fascia:ti,ab OR 'deep temporal fascia':ti,ab OR 'superficial temporal fascia':ti,ab OR variation:ti,ab OR variants:ti,ab OR variability:ti,ab OR morphology:ti,ab OR anatomy:ti,ab) AND Block C (imaging and clinical relevance): ('magnetic resonance imaging'/exp OR 'magnetic resonance imaging':ti,ab OR 'magnetic resonance':ti,ab OR 'ultrasonography'/exp OR ultrasonography:ti,ab OR ultrasound:ti,ab OR sonography:ti,ab OR orthognathic:ti,ab OR reconstructive:ti,ab OR surgery:ti,ab OR surgical:ti,ab) | (('temporalis muscle'/exp OR temporalis:ti,ab OR 'temporal muscle':ti,ab) AND (tendon:ti,ab OR aponeurosis:ti,ab OR fascia:ti,ab OR 'deep temporal fascia':ti,ab OR 'superficial temporal fascia':ti,ab OR variation:ti,ab OR variants:ti,ab OR variability:ti,ab OR morphology:ti,ab OR anatomy:ti,ab) AND ('magnetic resonance imaging'/exp OR 'magnetic resonance imaging':ti,ab OR 'magnetic resonance':ti,ab OR 'ultrasonography'/exp OR ultrasonography:ti,ab OR ultrasound:ti,ab OR sonography:ti,ab OR orthognathic:ti,ab OR reconstructive:ti,ab OR surgery:ti,ab OR surgical:ti,ab)) AND [english]/lim AND [humans]/lim | Apply [humans] and [english] limits. De-duplicate with MEDLINE results. No truncation or wildcards. |
